# Enzymatic Oxidation of Tea Catechins and Its Mechanism

**DOI:** 10.3390/molecules27030942

**Published:** 2022-01-29

**Authors:** Buhailiqiemu Abudureheman, Xiaochun Yu, Dandan Fang, Henghui Zhang

**Affiliations:** 1 College of Food Science and Engineering, Xinjiang Institute of Technology, Aksu 843000, China; buhalqam.a@163.com (B.A.); fdc2015087202112@126.com (D.F.); 2 College of Food Science and Engineering, Tonghua Normal University, Tonghua 134002, China; yuxc@thnu.edu.cn; 3 Department of Environment and Safety Engineering, Taiyuan Institute of Technology, Taiyuan 030008, China

**Keywords:** catechin, dimers, thearubigin, enzymatic oxidation

## Abstract

Tea (*Camellia sinensis*, *Theaceae*) is one of the most widely consumed beverages in the world. The three major types of tea, green tea, oolong tea, and black tea, differ in terms of the manufacture and chemical composition. Catechins, theaflavins, and thearubigins have been identified as the major components in tea. Other minor oligomers have also been found in tea. Different kinds of ring fission and formation elucidate the major transformed pathways of tea catechins to their dimers and polymers. The present review summarizes the data concerning the enzymatic oxidation of catechins, their dimers, and thearubigins in tea.

## 1. Introduction

Tea is one of the most widely consumed beverages in the world and is second only to water in popularity. The origin of tea has been traced back to the southern part of Yunnan Province in the southwest of China. More than 300 different kinds of tea are produced by different manufacturing processes. They are divided generally into three types: green tea (non-fermented), oolong tea (semi-fermented), and black tea (fermented). About 78% of the tea production worldwide is black tea, and green tea constitutes about 20% of tea production, consumed mainly in China and Japan. Oolong tea is partially fermented and constitutes about 2% of tea production. Catechins are the most abundant polyphenols in green tea. Black tea is manufactured by breaking the fresh leaves of *Camellia sinensis*. The main pigments in black tea are theaflavins and thearubigins, which are formed by the oxidation and polymerization of catechins during fermentation in the manufacturing process of tea. The chemistry information of theaflavins and their derivates has been elucidated perfectly. Although thearubigins account for up to 60% of the dry weight of black tea extract, the chemistry of thearubigins is still unclear. Thearubigins with simple structures have been elucidated. In the present review, we will discuss the current knowledge on the chemistry of catechins, their oxidation production derivates, and the major transformation pathways of them in tea.

## 2. Major Components in Tea

### 2.1. The Major Polyphenol Compounds in Green Tea

**Figure 1:**
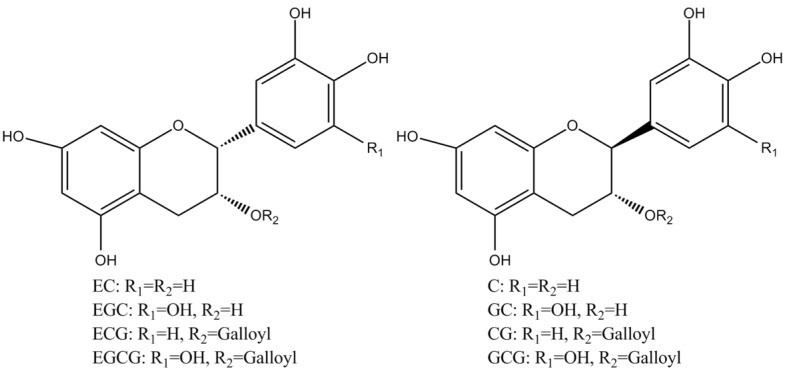
Structures of the major catechins in tea.

**Figure 2:**
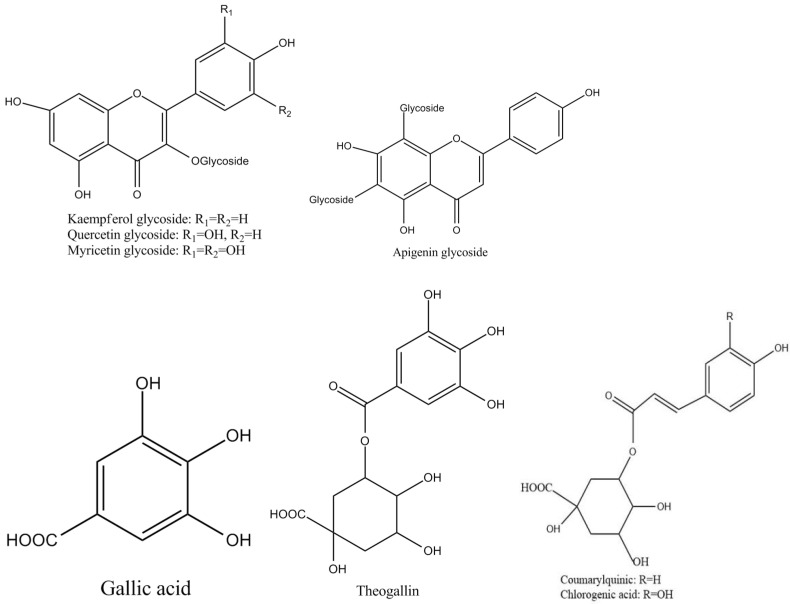
Structures of the major polyphenol compounds in tea.

Tea polyphenols, known as catechins, usually account for 30–42% of the dry weight of the solids in brewed green tea [1,2]. Catechins (flavan-3-ols) are the predominant form of flavonols in tea [1]. They are characterized by di-or tri-hydroxyl group substitution of the B ring and the meta-5,7-dihydroxy substitution of the A ring. The structures of the four major catechins, (−)-epigallocatechin gallate (EGCG), (−)-epigallocatechin (EGC), (−)-epicatechin gallate (ECG), and (−)-epicatechin (EC) are shown in Figure 1. EGCG is the major catechin in tea and accounts for 50–80% of the total catechins in tea [3]. In Figure 1, the racemic modifications of the four major cathechins as gallocatechin gallate (GCG), gallocatechin (GC), catechin gallate (CG), and catechin (C) are present in smaller quantities in tea [4]. Epicatechin digallate, epigallocatechin digallates, 3-methyl-EC, and 3-methyl-EGC have been identified in smaller quantities in tea [5]. Besides, 3″-methyl-EGCG, 4″-methyl-EGCG, and 4′,4″-di-methyl-EGCG have been identified in different tea species and at various seasons, ages of leaves, locations, and fermentation levels [6]. The flavonols such as kaempferol, quercitin, myricitin, and their glycosides have only been identified as significant components in tea. Flavones and their glycosides such as apigenin, the only flavone identified in tea, have also been detected in tea but represent a very small fraction of the tea polyphenols [7]. Gallic acid and its quinic acid ester, cinnamic acid derivatives of quinic acid, the coumaryl and caffeoyl-quinic acids, and chlorogenic acid have been identified in tea [4]. The major flavonols and flavones have been identified from green teas and fermented teas using liquid chromatography with diode array and electrospray ionization mass spectrometric detection (LC–DAD–ESI/MS), shown in Figure 2 [8].

### 2.2. Benzotroplone Derivates in Tea

Black tea is manufactured by plucking, withering, maceration (rolling), fermentation, and drying of the fresh leaves of *Camellia sinensis*. By withering, the leaves take on a form facilitating the maceration process, which results in disrupting the cell structure of the leaves. Then, the fermentation process begins. In this process, catechins (about 75%) contained in the tea leaves undergo enzymatic transformation consisting in oxidation and polymerization to yield a complex mixture of secondary polyphenols including theaflavins and thearubigins [9,10,11,12], theasinensins [13,14], theacitrins [15,16,17], and oolongtheanins [18] that contribute to the characteristic color and flavor of black tea. The resulting black tea composition depends on the technological process of its production. For the fermentation process, it is important to control the fermentation time and the oxidation environment such as temperature, humidity, and oxygen [19]. The concentrations of theaflavins and thearubigins increase as fermentation time increases, reaching optimum levels and then degrading if the fermentation time is prolonged. It is also necessary to control the environment for oxidation. During the fermentation of the ruptured tea leaves, in most cases, the process is performed at a temperature of 24–29 °C for 2–4 h or 55–110 min, under a high relative humidity of 95–98% with an adequate amount of oxygen.

There are two major enzymes involved in the fermentation process in making black tea [20]. One is polyphenol oxidase (PPO), which plays a key role in the oxidation of flavanols to theaflavins and thearubigins. The main effect of PPO is to oxidize catechins to theaflavins. Many studies have been carried out to study PPO-catalyzed formation of catechin oxidation products [21]. Peroxidase (POD) can also catalyze the oxidation of o-diphenols to their quinones in the presence of peroxide, such as hydrogen peroxide (H_2_O_2_), which are formed by the effect of PPO on certain flavanols. In fresh tea leaves, some POD activity is more than five times higher than that of PPO and has been found to increase during the manufacturing process of black tea [22]. At the beginning step of black tea processing, PPO is inhibited by heating, whereas POD remains active to a certain extent. Model oxidation systems have been used to compare the oxidation products obtained catalyzed with tea PPO and with horseradish POD [23]. However, the contribution of POD to the formation of black tea pigment in tea fermentation is still not entirely clear. Sang et al. have already studied the contribution of POD to the formation of theaflavins and thearubigins in an unnatural system [18,24].

**Figure 3:**
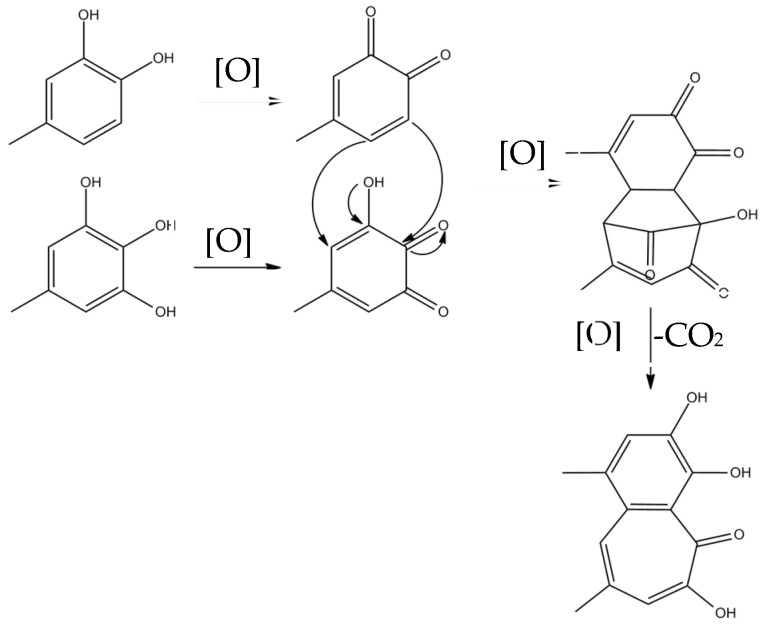
The formation pathway of theaflavins.

Theaflavins are orange or orange-red in color and possess a benzotropolone skeleton that is formed from the co-oxidation of selected pairs of catechins, one with a vic-trihydroxyphenyl moiety, and the other with an ortho-dihydroxyphenyl structure [18]. The primary step of catechin oxidation is believed to be the overall conversion of the ortho-dihydroxy (‘catechin’) and ortho-trihydroxy (‘gallocatechin’)-phenyl ‘B’ rings to give the corresponding highly reactive orthoquinones. The formation of theaflavins is between the corresponding two quinonoid species. Then, the formation of the benztropolone group requires the loss of one carbon atom as carbon dioxide. The formation pathway of theaflavins is shown in Figure 3. The oxidative coupling of two catechins is dependent on structural and redox potential factors. In many studies concerning the enzymatic oxidation of tea catechins, oxidative coupling reactions of catechin B-rings have been demonstrated [13,25]. The redox potential of the galloyl group is higher than that of B rings and its reactivity with o-quinones is comparatively low. A total of 60–80% of the total tea catechins possess galloyl esters located at the C-3 hydroxy group and oxidation of galloyl groups may be important to the formation of black tea pigment [26]; only limited examples of oxidative coupling of galloyl groups have been reported [17,27,28,29]. Besides, enzymes preferentially oxidize the catechol B-rings, and the resulting quinone subsequently oxidizes the pyrogallol rings for the redox potential of which is lower than that of the catechol rings [30,31,32,33].

**Figure 4:**
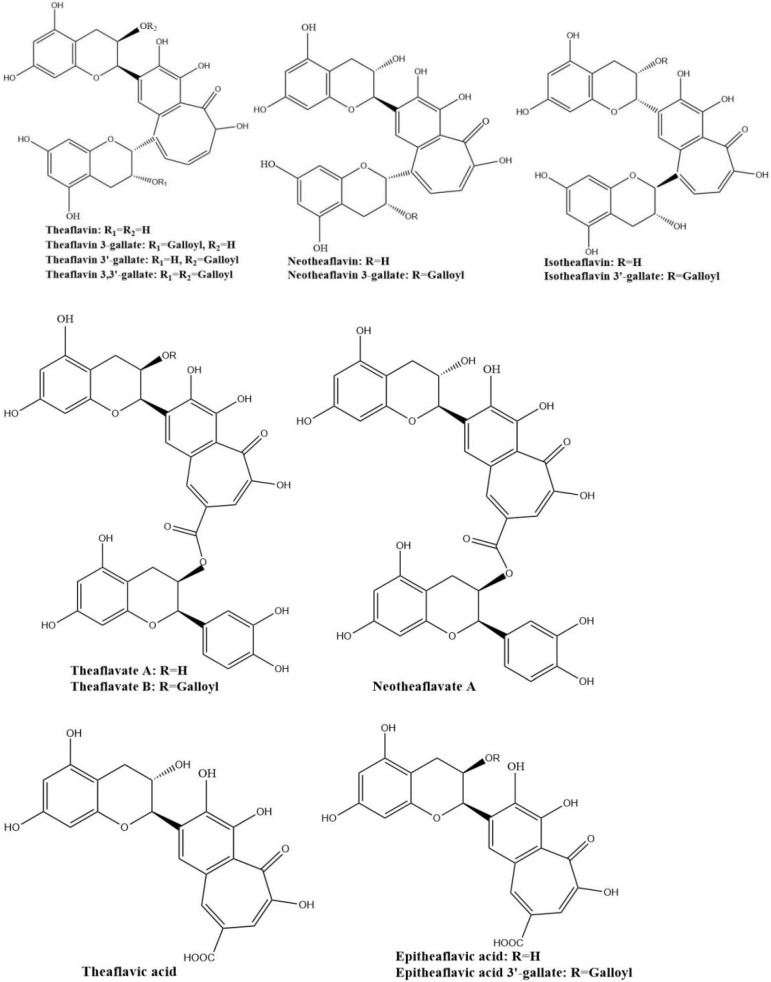
Structures of the major theaflavin derivates in black tea.

The four major theaflavins are theaflavin, theaflavin 3-gallate, theaflavin 3′-gallate, and theaflavin 3,3′-digallate, which are formed by coupling between EC and EGC, EC and EGCG, EGC and EGC, and EGC and EGCG, respectively. Stereoisomers of theaflavins and their closely related benzotropolone compounds such as neo-theaflavins, iso-theaflavins, theaflavate, theaflavic acids, and methylated theaflavins, etc., have also been identified from black tea [31,32,33,34]. They are shown in Figure 4. Under the catalysis of PPO and POD, neotheaflavin was formed using a monitored reaction between C and EGC. In the neotheaflavin family, neotheaflavin 3-gallate was isolated from black tea and structurally characterized using NMR and MS [35,36]. Neotheaflavin 3-gallate was formed by coupling between C and EGCG [37]. Isotheaflavin is formed hypothetically to couple between EC and GC. The total concentration of isotheaflavin in black tea is too small and it has not been detected. Isotheaflavin 3′-gallate has been characterized in extracts from black tea and its structure was determined using NMR spectroscopy. The proposed formation of isotheaflavin 3′-gallate is by coupling between EC and GCG [38]. Theaflavates have been found in some black tea extracts and enzymatically formed. Theaflavate A gets a novel benzotropolone skeleton formed between the B-ring of one ECG molecule and the galloyl ester group of another [39]. Theaflavate B is between the B-ring of an EC molecule and the galloyl ester group of ECG [40]. Neotheaflavate B was formed between the B-ring of one C molecule and the galloyl ester group of ECG using horseradish POD in the presence of H_2_O_2_ [35]. The reactions of EC and ECG with gallic acid in a model tea fermentation system were studied. The primary oxidation products formed from the oxidation of EC and ECG with gallic acid in short reaction periods were epitheaflavic acid and 3-galloyl epitheaflavic acid. Theaflavic acid has been found in some black tea extracts and enzymatically formed by the reaction of C and gallic acid along with purpurogallin carbolic acid [41]. In the model tea fermentation system, epitheaflavic acid and epitheaflavic acid 3-gallate were formed by the reactions of EC and ECG with gallic acid [42]. Epitheaflavic acid was rapidly transformed to thearubigins in the presence of EC, which shows the possible mechanism of the thearubigin formation [42]. These theaflavin derivates are usually minor components or hardly detectable in black tea compared with the four major theaflavins. Due to their limited availability, the biological properties of these theaflavin derivates have been barely evaluated.

**Figure 5:**
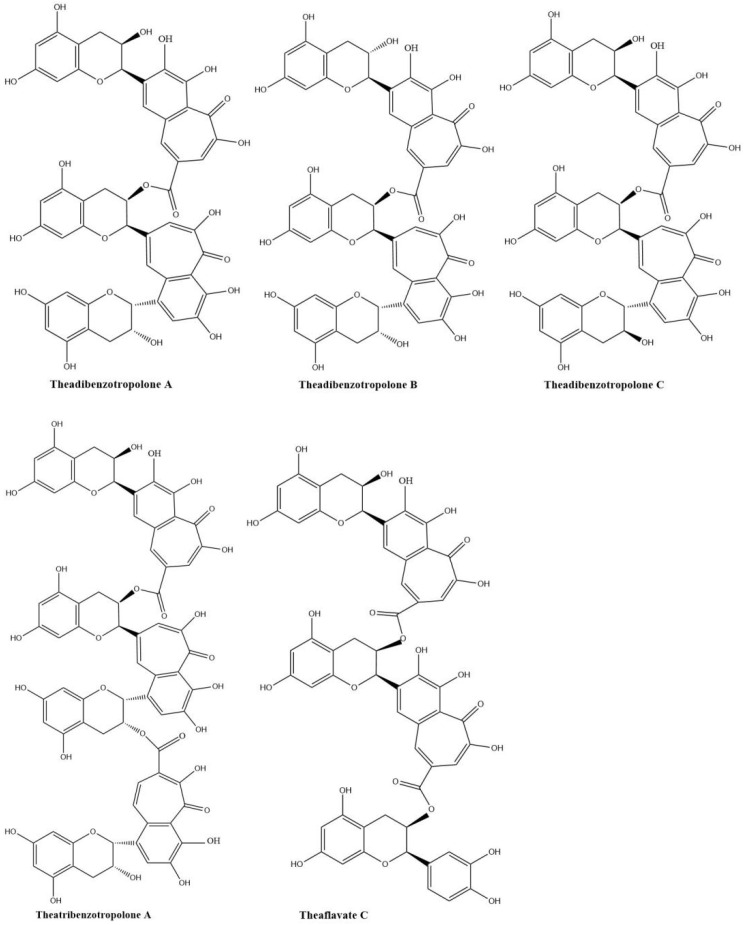
Structures of theadibenzotropolone A, B, and C; theatribenzotropolone A; and theaflavate C.

Sang et al. (2004) already synthesized eighteen benzotropolone derivatives, which include all the major theaflavins, theaflavates, and theaflavic acids reported in black tea as well as several new benzotropolone derivatives by the reaction of selected pairs of catechins using horseradish POD in the presence of H_2_O_2_ [18]. They also found theaflavins can further react with tea catechins to form di- or tri-benzotropolone-type compounds. The galloyl ester group of theaflavins is as reactive as the B-ring (vic-trihydroxy) of EGCG or EGC and can be oxidized to form di- or tri-benzotropolone skeletons, strongly implying that this mechanism is an important pathway to extend the molecular size of thearubigins. The galloyl ester group of theaflavin 3-gallate can further react with EC to form the new theaflavin type tea catechin trimer, theadibenzotropolone A, which was characterized from black tea extract by LC/ESI–MS/MS [21,43]. Theaflavin 3-gallate can react with EC to form theadibenzotropolone B, the isomer of theadibenzotropolone A. Neotheaflavin 3-gallate could react with C to form theadibenzotropolone C (Figure 5). However, theaflavin 3-gallate could not react with C to form the isomer of theadibenzotropolone. Interestingly, theaflavin 3,3′-digallate are not expected to react with EC or C to form the two isomers of theadibenzotropolone. However, theatribenzotropolone A was obtained by the reaction between theaflavin 3,3′-digallate and EC. The existence of these compounds in black tea was characterized by tandem mass spectrometry (MS/MS) through collision-induced dissociation (CID) [44]. Theaflavate C is a trimer of ECG and possesses two benzotropolone moieties formed between the B-ring of one ECG molecule and the galloyl ester group of another [40].

### 2.3. Theanaphthoquinone in Tea

**Figure 6:**
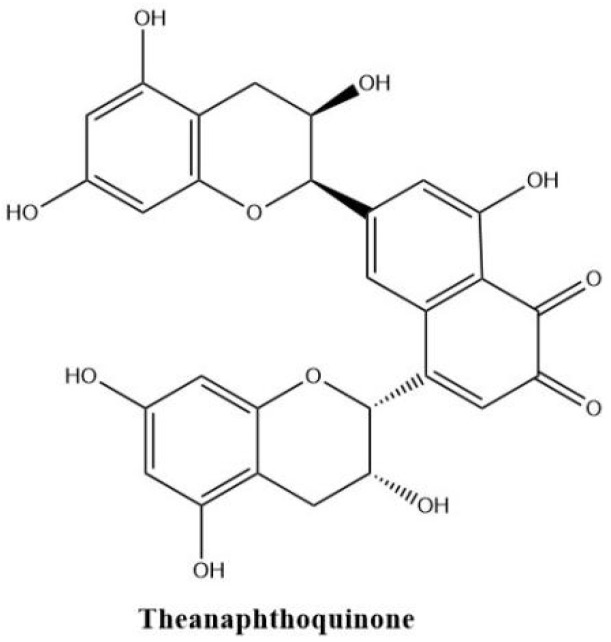
The structure of theanaphthoquinone.

Theanaphthoquinone in Figure 6 gets a 1, 2-naphthoquinone moiety oxidatively derived from the benzotropolone unit of theaflavin. Theanaphthoquinone was generated by the treatment of a mixture of EC and EGC with fresh tea leaf or banana fruit homogenate. It is proposed that theanaphthoquinone is biosynthesized from theaflavin with the aid of PPO [45].

### 2.4. Theaflagallinas in Tea

**Figure 7:**
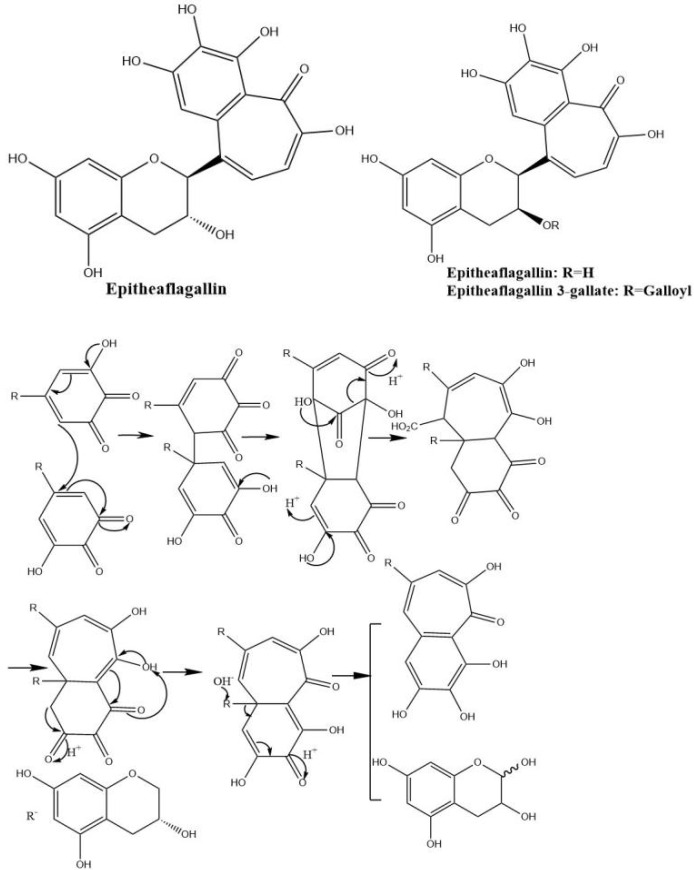
The structures of theaflagallinas and proposed mechanisms for production of epitheaflagallin.

Theaflagallins having a characteristic 1′,2′,3′-trihydroxy-3,4-benzotropolone unit are produced by condensation between two pyrogallol rings which were identified in black tea and enzymatically formed by the oxidation of catechins and pyrogallol. Theaflagallin, epitheaflagallin, and epitheaflagallin-3-gallate are the three major theaflagallins. They can be formed from the reaction of pyrogallol with C, EC, and EGC [46]. However, pyrogallol itself does not occur in fresh tea leaf, so it is proposed that theaflagallins are not formed by the oxidation of catechins and pyrogallol in tea fermentation. It was revealed that epitheaflagallin was produced from EGC alone even in the absence of gallic acid. The key intermediate of epitheaflagallin formation in tea is shown in Figure 7. Migration or elimination of the C-ring occurs and flavan A is produced and isolated from black tea [26].

### 2.5. Theasinensin Derivates and Oolongtheanins in Tea

**Figure 8:**
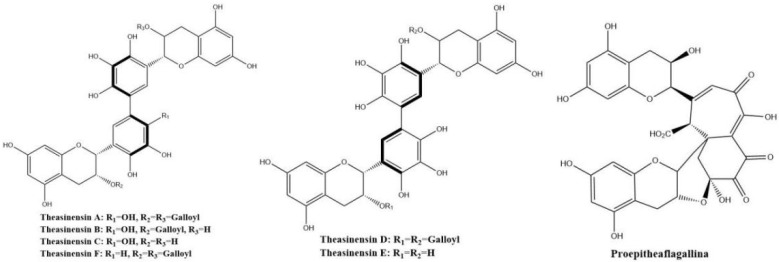
Structures of theasinensins and proepitheaflagallina.

Theasinensins shown in Figure 8 present mainly in oolong tea represent a new class of dimeric gallocatechins linked by C–C bonds between the two ‘B’ rings, forming a biphenyl grouping. Theasinensin A (EGCG dimer), B (EGCG and EGC dimer), C (EGC dimer), and F (EGCG and ECG dimer) are the most abundant ones [47].

**Figure 9:**
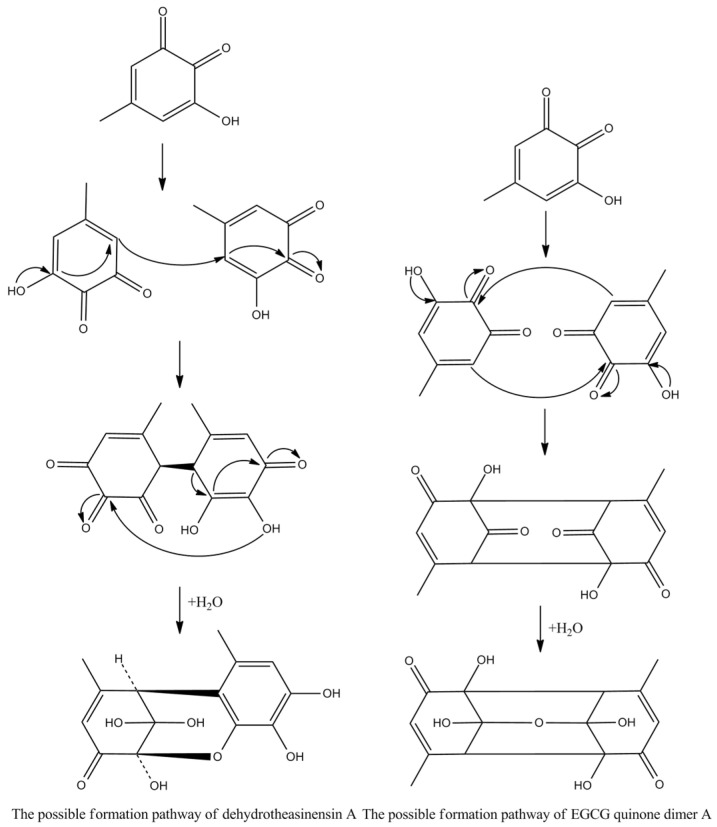
The possible mechanism for the formation of dehydrotheasinensin A and EGCG quinone.

Theasinensins A and D are B,B′-linked dimers of EGCG connected through R and S biphenyl bonds, respectively. Theasinensin A has been identified as the major oxidation product of EGCG under cell culture conditions as well [48]. Enzymatic oxidation of EGCG with a Japanese pear homogenate produced dehydrotheasinensin A and EGCG quinone dimer A. The possible mechanism for the formation of dehydrotheasinensin A and EGCG quinone dimer A are shown in Figure 9. The reduction in dehydrotheasinensin A with ascorbic acid or thiol compounds yielded theasinensin A. When the aqueous solution of dehydrotheasinensin A was heated, theasinensin D was produced along with galloyl oolongtheanin. On the other hand, dehydrotheasinensin A was converted to theasinensins A and D along with galloyl oolongtheanin in phosphate buffer at pH 6.8 at room temperature [49]. Oxidation of EGC with a Japanese pear homogenate gave dehydrotheasinensin C, proepitheaflagallina in Figure 8, and an EGC quinone dimer. The possible mechanism for the formation of dehydrotheasinensin C and EGC quinone dimer is the same to that of dehydrotheasinensin A and EGCG quinone dimer A. Dehydrotheasinensin C has a hydrated cyclohexenetrione structure and its oxidation–reduction dismutation reaction yielded theasinensins C and E, and desgalloyl oolongtheanin [26]. On hydrogenation with dithiothreitol, dehydrotheasinensin C was converted to theasinensin C [26]. In neutral phosphate buffer, dehydrotheasinensin C was decomposed to give theasinensin C, theasinensin E, desgalloyl oolongtheanin, and dehydrotheasinensin E [13]. Proepitheaflagallina was degraded into epitheaflagallin and epitheaflagallin by heating at 80 °C for 10 min in an aqueous solution. Recently, it was found that unripe fruit homogenate of Citrus unshiu selectively oxidizes pyrogallol-type catechins to yield only dehydrotheasinensins [50]. The selectivity of unripe fruit homogenate of Citrus unshiu is probably related to the lower redox potential of pyrogallol-type catechins.

Oolongtheanins-desgalloyl oolongtheanin, oolongtheanin, and galloyl oolongtheanin were originally identified as the polyphenols contained in oolong tea and black tea [50]. Oolongtheanins coexist with theasinensins and they are the oxidation–reduction products of dehydrotheasinensins and are related to the oxidation–reduction dismutation of dehydrotheasinensins. The structure of desgalloyl oolongtheanin was revised by Yosuke Matsuo et al. based on the spectroscopic and computational data collected in the current study, and a mechanism responsible for the production of oolongtheanins is also proposed [50].

### 2.6. Theacitrin Derivates in Tea

**Figure 10:**
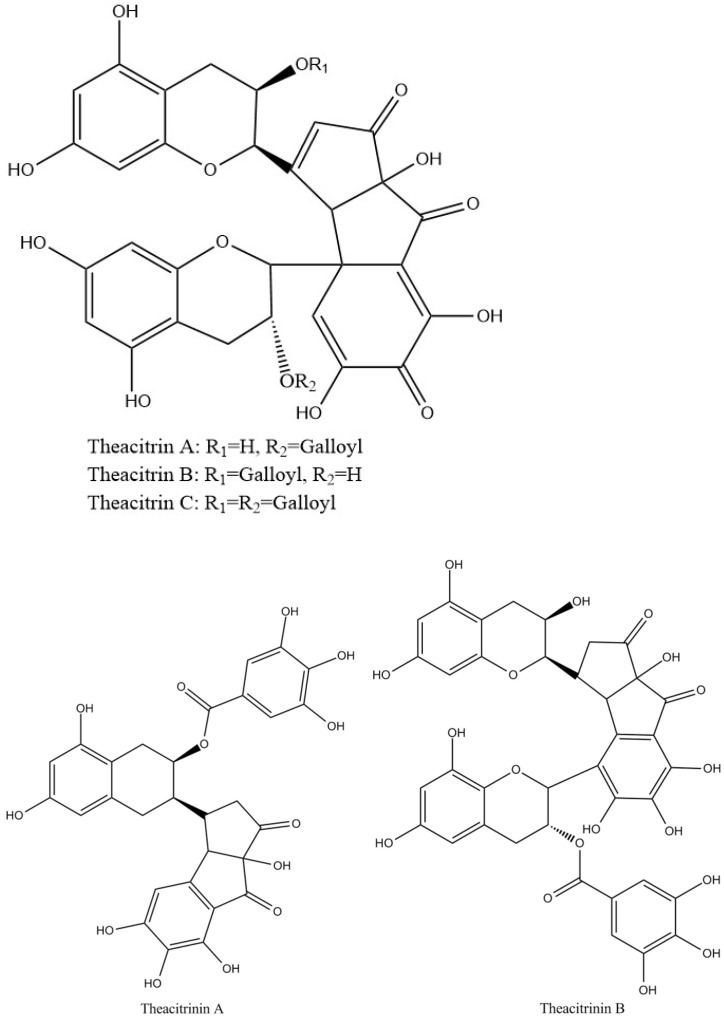
Structures of theacitrinin A, B, and theacitrins.

**Figure 11:**
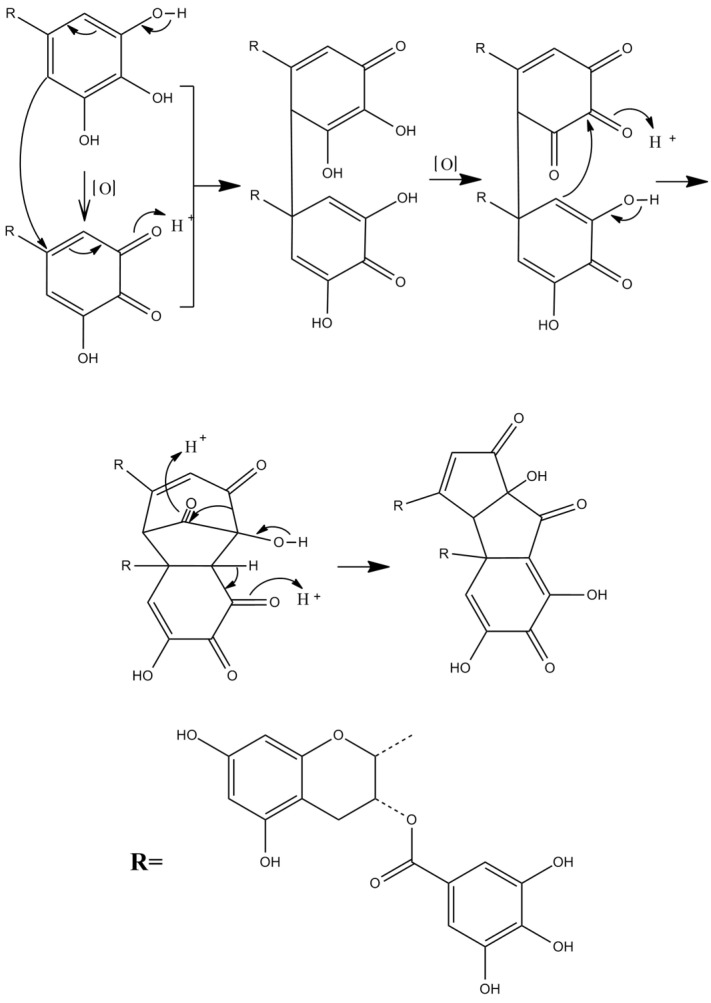
The possible mechanism for the formation of theacitrin C.

Theacitrins are yellow compounds isolated from the thearubigin fractions of an Assam black tea and their preliminary structural data have been reported [51]. Theacitrins are found to be highly unstable but their structures have been elucidated successfully. Theacitrin A and B are dimers of EGCG and EGC. Theacitrin C is dimer of EGCG. Theacitrin A, B, and C are shown in Figure 10. The separation, purification, and characterization of theacitrin A have been characterized unequivocally by Davis et al. [51]. Oxidation of EGCG with a Japanese pear homogenate gave theacitrin C. The B-ring of EGCG is oxidized to its o-quinone form, and 1,4-addition then occurs to generate a C–C bond. Successive oxidation and intramolecular 1,2-addition produces a bicyclo octane-type intermediate, which subsequently rearranges to afford theacitrin C [52]. The possible mechanism for the formation of theacitrin C is shown in Figure 11. Degradation of theacitrin C by heating at 80 °C for 60 min in an aqueous solution gets the decomposition products detected as a broad hump on the HPLC baseline. However, heating in an aqueous solution containing 0.1% TFA theacitrin C was degraded into theacitrinin A and 2,3,5,7-tetrahydroxychroman-3-*O*-gallate. Although theacitrinin A was not isolated from black tea produced in India and Sri Lanka at the present stage, theacitrinin B has been isolated from black tea whose ^1^H and ^13^C NMR spectra were closely related to those of theacitrinin A, except for the appearance of signals attributable to one set of flavan A-and C-rings and one galloyl group. Besides, the proposed mechanism of theacitrinin B production from theacitrin A has been put forward [53,54].

### 2.7. Dimers of Theaflavin Derivates in Tea

**Figure 12:**
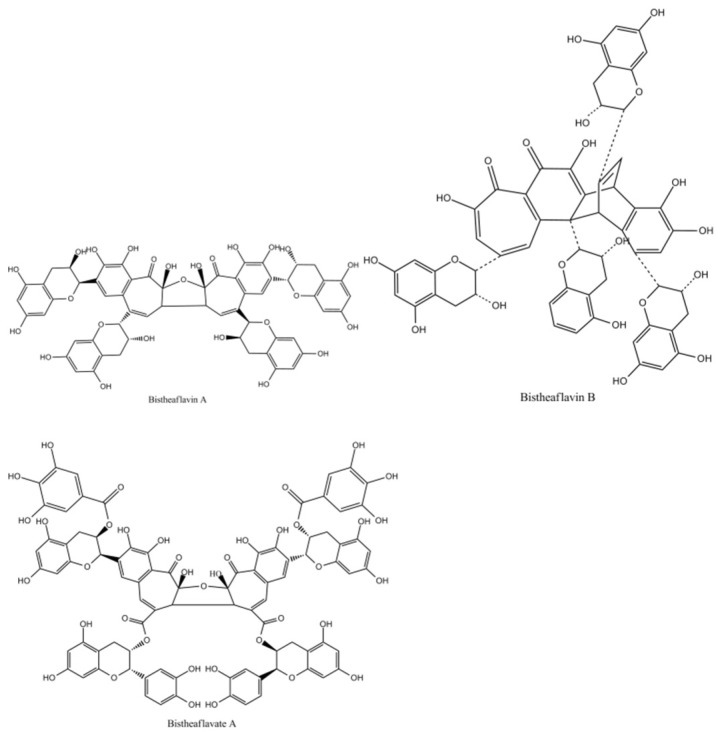
The Structures of bistheaflavate A, B, and theaflavate C.

Bistheaflavins A and B in Figure 12 are two theaflavin oxidation products. Treatment of a mixture of EC and EGC with banana fruit homogenate yielded bistheaflavin A together with theaflavin and theanaphthoquinone. Bistheaflavin A was formed by oxidative C–C coupling of two theaflavin molecules. In contrast, theaflavin in phosphate buffer (pH 7.3) was gradually oxidized to give bistheaflavin B and theanaphthoquinone. Bistheaflavin B possesses a bicyclooctane skeleton probably formed by intermolecular cyclization between dehydrotheaflavin and dihydrotheanaphthoquinone [51].

Enzymatic oxidation of ECG yielded bistheaflavate A in Figure 12, along with theaflavate A, a known dimer of ECG generated by coupling of the B-ring with the galloyl group. Bistheaflavate A was a tetramer produced by intermolecular coupling of two benzotropolone moieties of theaflavate A. From its structure, it was deduced that oxidative coupling of galloyl groups resulted in extension of the molecular size of the products in catechin oxidation [55].

### 2.8. Thearubigins in Tea

Thearubigins, red-brown or dark brown, which comprise about 20% (*w*/*w*) of extracted solids, are heterogeneous polymers of tea catechins [56]. Information of their formation and structures is still very limited. Kuhnert (2010) showed that during black tea manufacture, fresh tea leaf catechins are oxidized to ortho-quinones. These react with a nucleophile, either water to form oxygenated catechins, with another catechin to form dimeric theasinensins, theaflavins, oolongtheanins, theanaphthoquinones or theacitrins, or higher catechin oligomers. Highly oxygenated black tea polyphenols are subject to further oxidation to form quinone and quinone-methide type derivatives, which are in equilibrium with their reduced counterparts present within the black tea infusion all together accounting for around 95% of the thearubigins constituents observed.

There are several classifications of thearubigins. The first one classifies thearubigins into three groups in terms of their solubility in different solvents. Thearubigins of SI type can be extracted into ethyl acetate, whereas SIa and SII type remain in the aqueous phase and SIa are more soluble in diethyl ether than the aqueous phase [39]. Another classification method is based on the chromatographic behavior of thearubigins in Hypersil ODS. Group I runs close to the void volume of the columns; group II is resolved thearubigins; and group III is unresolved thearubigins [57]. In recent years, with the assistance of modern advanced instrumental analysis technology, identification and characterization of thearubigins in black tea have progressed further. The formation of oligomeric thearubigins from catechins, theaflavins, theanaphthoquinone, theasinensins, theacitrins, and oolongtheanins has been suggested.

A previous study using chromatography and chemical degradation of isolated fractions and a possible partial structure of polymeric thearubigins from black tea was elucidated using chemical degradation, determining that they are heterogeneous polymers of flavan-3-ols and flavan-3-ol gallates having bonds at C-4, C-6, C-8, C-2′, C- 5′ and C-6′ in the flavan-3-ol units [24]. Epitheaflavic acid was rapidly transformed to thearubigins in the presence of EC, suggesting the possible mechanism of the thearubigin formation. A prolonged experiment with tea leaf extract showed a decrease in theaflavin and theanaphthoquinone and an increase in polymeric substances suggesting that theanaphthoquinone was further transformed during tea fermentation and might be related to the formation of thearubigin [58]. Theaflavins further react with tea catechins to form di- or tri-benzotropolone-type compounds strongly implying that this mechanism is an important pathway to extend the molecular size of thearubigins [21]. Using delayed pulsed ion extraction of ions generated via the matrix-assisted laser desorption ionization (MALDI) technique, on line with a linear time-of-flight (TOF) mass spectrometer, Sang et al. found that theasinensins and procyanidins could also react with catechins to generate benzotropolone-type polymers [13]. Kuhnert et al. (2010), relying on LC/MS/MS, elucidated that thearubigins are solely composed from low molecular weight compounds with a mass below 2100 g/mol and revealed many thearubigin structures and valuable chemical information. In addition, Kuhnert et al. lead to a novel hypothesis for the formation and structure of the black tea thearubigins named oxidative cascade hypothesis [16]. Using ESI/HPLC tandem mass spectrometry in the SII fraction of black tea thearubigins, two novel homologous series of polyhydroxylated theasinensins and theanaphthoquinones were revealed which corresponded to the prolonged experiment treating a mixture of EC and EGC with tea leaf extract. The first homologous series of compounds revealed the presence of polyhydroxylated dimers of the theanaphthaquinone and theasinensin C structures. In addition, new classes of peroxo-/epoxy- compounds in the series of theasinensin A were identified, which indicated the presence of H_2_O_2_ and its important contribution as a nucleophile in the tea fermentation process [58].

Since thearubigins were first introduced fifty years ago, much of the thearubigin structures and valuable chemical information have been elucidated. Up to now, the chemical nature of thearubigins remains largely unresolved. Much more information about thearubigins, including structure formation and conformation, isolation of single compounds and their characterization, evaluation of their contribution to taste, and knowledge of biological properties, needs to be studied further.

## 3. Conclusions

In summary, tea is one of the most consumed functional beverages in the world. It contains large amounts of polyphenols including catechins, their dimers, and thearubigins. Various tea polyphenols such as isotheaflavin and neotheaflavin are usually minor components or hardly detectable in tea. The biological properties of tea polyphenol have been scarcely evaluated. The model study of tea fermentation has been carried out to elucidate the structure, isolation, characterization, and biological property of tea polyphenols. Thearubigins isolated from a typical tea fermentation comprise very closely related structures, which are solely composed from low molecular weight compounds with a mass below 2100 g/mol. Thearubigins range from trimeric to tetrameric structures, and possibly greater. The formation of oligomeric thearubigins in a model study of tea fermentation consumes dimeric catechins which partially consist of productions of thearubigin degradation. The way to extend the molecular weight of theaflavin derivates suggests a possible mechanism extending the molecular size of thearubigins. The existing information about thearubigins is valuable but further study is required.
